# Identification of Key Prognostic Biomarker and Its Correlation with Immune Infiltrates in Pancreatic Ductal Adenocarcinoma

**DOI:** 10.1155/2020/8825997

**Published:** 2020-08-31

**Authors:** Hong Luan, Chuang Zhang, Tuo Zhang, Ye He, Yanna Su, Liping Zhou

**Affiliations:** ^1^Department of Laboratory Medicine, The First Affiliated Hospital of China Medical University, Shenyang, Liaoning 110001, China; ^2^Key Laboratory of Anticancer Drugs and Biotherapy, The First Affiliated Hospital of China Medical University, Shenyang, Liaoning 110001, China; ^3^Department of Medical Oncology, The First Affiliated Hospital of China Medical University, Shenyang, 110001 Liaoning, China; ^4^Department of Graduate Faculty, The First Affiliated Hospital of China Medical University, Shenyang, Liaoning Province 110001, China

## Abstract

Pancreatic ductal adenocarcinoma (PDAC) is an extremely malignant tumor. The immune profile of PDAC and the immunologic milieu of its tumor microenvironment (TME) are unique; however, the mechanism of how the TME engineers the carcinogenesis of PDAC is not fully understood. This study is aimed at better understanding the relationship between the immune infiltration of the TME and gene expression and identifying potential prognostic and immunotherapeutic biomarkers for PDAC. Analysis of data from The Cancer Genome Atlas (TCGA) and the Gene Expression Omnibus (GEO) databases identified differentially expressed genes (DEGs), including 159 upregulated and 53 downregulated genes. Gene Ontology analysis and Kyoto Encyclopedia of Genes and Genomes enrichment were performed and showed that the DEGs were mainly enriched for the PI3K-Akt signaling pathway and extracellular matrix organization. We used the cytoHubba plugin of Cytoscape to screen out the most significant ten hub genes by four different models (Degree, MCC, DMNC, and MNC). The expression and clinical relevance of these ten hub genes were validated using Gene Expression Profiling Interactive Analysis (GEPIA) and the Human Protein Atlas, respectively. High expression of nine of the hub genes was positively correlated with poor prognosis. Finally, the relationship between these hub genes and tumor immunity was analyzed using the Tumor Immune Estimation Resource. We found that the expression of SPARC, COL6A3, and FBN1 correlated positively with infiltration levels of six immune cells in the tumors. In addition, these three genes had a strong coexpression relationship with the immune checkpoints. In conclusion, our results suggest that nine upregulated biomarkers are related to poor prognosis in PDAC and may serve as potential prognostic biomarkers for PDAC therapy. Furthermore, SPARC, COL6A3, and FBN1 play an important role in tumor-related immune infiltration and may be ideal targets for immune therapy against PDAC.

## 1. Introduction

Pancreatic ductal adenocarcinoma (PDAC) is an extremely malignant tumor of the digestive system, with a 5-year survival rate of only 8% [1]. GLOBOCAN 2018 estimated that pancreatic cancer ranked as the seventh leading cause of cancer death worldwide, with approximately 458,918 new incidence cases and 432,242 deaths [2]. Because of its poor prognosis, newly diagnosed cases and deaths from pancreatic cancer have significantly increased at the same pace over the last few decades [2]. Currently, surgical resection is still the most effective treatment, which significantly improves the 5-year survival rate to 20–30%. However, less than 20% of all patients are eligible for resection because the majority of patients have locally advanced or metastatic disease at the time of diagnosis [3]. Therefore, there is an urgent need for more effective therapeutic strategies.

In recent years, cancer immunotherapy has become an active area of cancer research. This approach is designed to improve the immunogenicity of tumor cells, stimulate and enhance the antitumor immune response, and inhibit tumor growth and progression. Since the FDA approval of the first targeted drug ipilimumab (humanized anti-CTLA-4 IgG1 monoclonal antibody) in 2011, six more immune checkpoint inhibitors have been approved for cancer therapy [4, 5]. At present, immune checkpoint inhibitors have achieved significant therapeutic effects in melanoma [6], squamous non-small-cell lung cancer [7], renal cell carcinoma [8, 9], and urothelial cancer [10, 11]. However, the immune profile of PDAC and the immunologic milieu of its tumor microenvironment (TME) are unique relative to other malignant tumors, and the mechanism of how the TME engineers the carcinogenesis of PDAC is not entirely clear. Therefore, it is necessary to find new possible prognostic and immunotherapeutic biomarkers for this disease.

In the current study, we analyzed pancreatic cancer gene expression data from The Cancer Genome Atlas (TCGA) and Gene Expression Omnibus (GEO) databases to identify differentially expressed genes (DEGs). Gene Ontology (GO) and Kyoto Encyclopedia of Genes and Genomes (KEGG) pathway enrichment and protein-protein interaction (PPI) network analyses were performed to reveal the interactive relationships between the DEGs to explore the underlying molecular mechanisms involved in the carcinogenesis and progression of PDAC. Subsequently, we used the cytoHubba plugin of Cytoscape to search for hub genes and identify the most significant hub genes using four different models (Degree, Maximal Clique Centrality (MCC), Density of Maximum Neighborhood Component (DMNC), and Maximum Neighborhood Component (MNC)). We further validated the expression levels of the hub genes and their clinical relevance using Gene Expression Profiling Interactive Analysis (GEPIA) and the Human Protein Atlas, respectively. Finally, we evaluated the associations between the prognosis-related genes and several immune cell types based on the Tumor Immune Estimation Resource (TIMER). In summary, our study identified potential prognostic biomarkers and immunotherapeutic targets for PDAC.

## 2. Materials and Methods

### 2.1. Data Collection and Processing

The mRNA expression profiles of PDAC and adjacent normal tissues were obtained from GEPIA (TCGA Data Online Analysis Tool; http://gepia2.cancer-pku.cn/#index). PDAC microarray profiles were retrieved from the GEO database (http://www.ncbi.nlm.nih.gov/geo/) using the keywords “pancreatic cancer” OR “PDAC” OR “pancreatic adenocarcinoma.” “Homo sapiens” and “Expression profiling by array” were included in the next round of screening. Inclusion criteria consisted of (1) human pancreatic tissue samples, (2) tumor and adjacent noncancerous tissue samples, and (3) more than 30 samples in the tumor and adjacent noncancerous groups. Based on these criteria, the gene expression microarray datasets GSE15471 [12] and GSE62452 [13] were downloaded for analysis. GSE15471 is comprised of 39 tumors and paired adjacent normal tissues, and GSE62452 contains 69 tumors and 61 adjacent normal tissue samples. We used the GEO2R analysis tool (http://www.ncbi.nlm.nih.gov/geo/geo2r/) to screen for differentially expressed mRNAs between the normal tissue and tumor samples from the GSE15471 and GSE62452 datasets. *P* < 0.05 and ∣log2FC | >1 were set as the cut-off criteria for identifying DEGs.

### 2.2. Functional Enrichment Analysis

Gene Ontology (GO) analysis and Kyoto Encyclopedia of Genes and Genomes (KEGG) enrichment were performed on the DEGs, and the results were visualized using the R package. A *P* value < 0.05 was set as the cut-off criterion.

### 2.3. PPI Network Construction and Module Analysis

The STRING database (https://string-db.org/) was used to draw the PPI network diagram of the DEGs. The hub genes were identified and visualized using Cytoscape [14]. Specifically, the hub genes were identified using the Degree, MCC, DMNC, and MNC models with the Cytoscape plugin cytoHubba [15].

### 2.4. Validation of Hub Genes

We analyzed the expression of the hub gene using GEPIA (http://gepia.cancer-pku.cn/index.html) [16]. Because there were few normal pancreatic tissue samples in the TCGA database, the expression level of specific DEGs was validated using the TCGA PDAC tumor data and matched data for normal tissue in the TCGA and Genotype-Tissue Expression (GTEx) databases. ∣log2FC | >1 and *P* < 0.05 were considered statistically significant. Protein expression of the DEGs was evaluated in the pancreatic tumor and nontumor tissues using the Human Protein Atlas tool (https://www.proteinatlas.org/) [17]. Mutation data were obtained from the cBioPortal for Cancer Genomics (https://www.cbioportal.org/) [18].

### 2.5. Association of Hub Gene Expression with the Survival of Patients with PDAC

We analyzed the association of patient prognosis with the hub genes using the human protein atlas website. Based on the fragments per kilobase million (FPKM) value of each gene, pancreatic cancer patients were classified into two expression groups (high expression and low expression), and the correlation between the expression level and patient survival was examined. The prognosis of each group of patients was examined using Kaplan-Meier survival estimators, and the survival outcomes of the two groups were compared by the log-rank test. *P* < 0.05 was considered statistically significant.

### 2.6. Associations between Hub Genes and Immune Cell Infiltration and Immune Checkpoints

TIMER is an online website that uses RNA-Seq expression profiling data to detect immune cell infiltration in more than 30 cancer types [19]. We used this approach to analyze the correlation between the expression levels of the hub genes and the abundance of immune infiltrates (B cells, CD4+ T cells, CD8+ T cells, neutrophils, macrophages, and dendritic cells) and four immunological checkpoints (CTLA4, CD274, PDCD1, and PDCD1LG2).

## 3. Results

### 3.1. Screening for DEGs in PDAC

**Figure 1:**
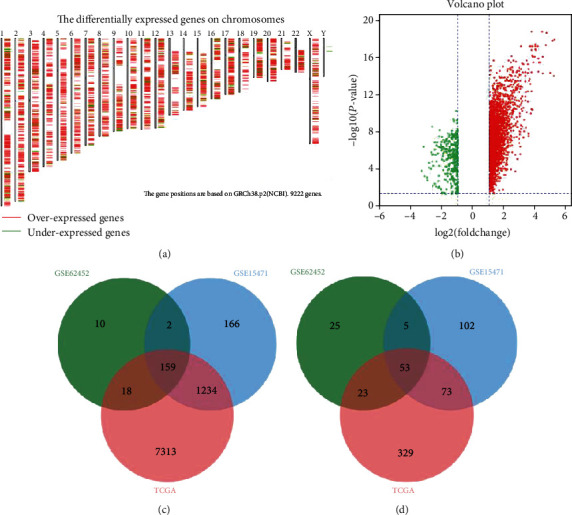
Identification of DEGs in pancreatic cancer using three databases. (a) The differentially expressed genes from the TCGA database were determined by GEPIA. (b) The volcano map for the GSE15471 dataset. (c) A Venn diagram showing 159 upregulated genes in PDAC. (d) A Venn diagram showing 53 downregulated genes in PDAC.

We identified the differentially expressed genes in PDAC in the TCGA database using GEPIA (Figure 1(a)). Based on the thresholds of *P* < 0.05 and |log2FC | >1, a total of 9202 DEGs (8724 upregulated and 478 downregulated) were identified. DEGs between normal and cancer tissues in the GEO datasets GSE15471 and GSE62452 were screened using GEO2R. A total of 295 DEGs (189 upregulated and 106 downregulated genes) were identified in profile GSE62452, and 1794 DEGs (1561 upregulated and 233 downregulated genes) were found in profile GSE15471 (Figure 1(b)). The overlapping DEGs within the three datasets consisted of 159 upregulated genes (Figure 1(c)) and 53 downregulated genes (Figure 1(d)).

### 3.2. Functional Analysis of the DEGs

**Figure 2:**
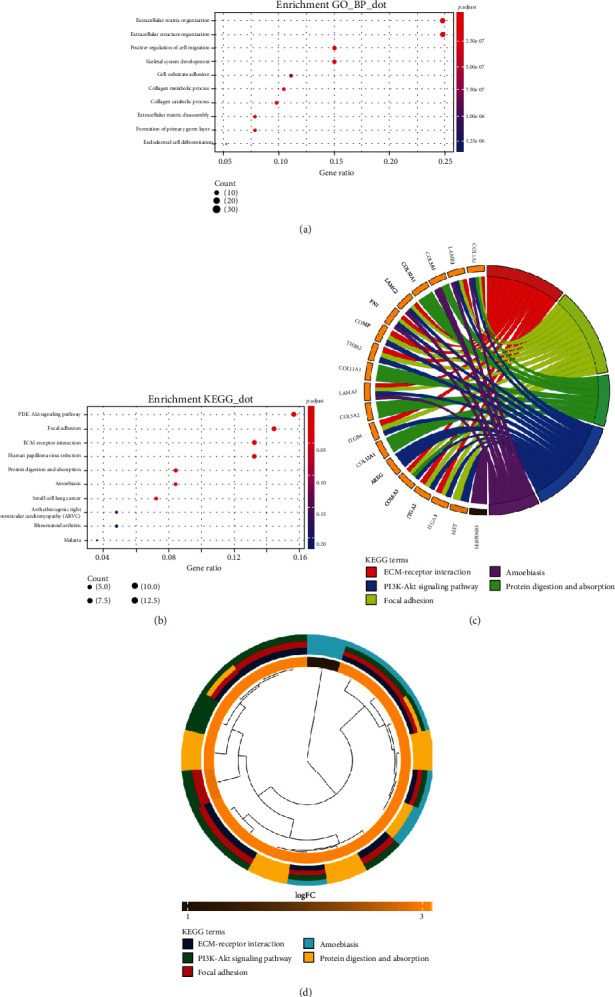
Functional enrichment analysis of DEGs. (a) Top ten enriched biological processes for the DEGs. (b) Top ten enriched KEGG pathways for the DEGs. (c) Hierarchical clustering of gene expression profiles in each KEGG pathway. (d) Chord plots showing the relationship between the hub genes and the KEGG pathway.

To analyze the underlying interplay of the DEGs, GO analysis and KEGG pathway enrichment were performed using the R package. Based on the GO analysis, the upregulated DEGs mainly participated in the extracellular matrix organization, extracellular structure organization, positive regulation of cell migration, skeletal system development, and cell-substrate adhesion (Figure 2(a)). KEGG pathway analysis revealed that the DEGs were mainly enriched in the PI3K-Akt signaling pathway, focal adhesion, and ECM-receptor interaction (Figure 2(b)). Genes specifically enriched in each KEGG term were visualized (Figures 2(c) and 2(d)).

### 3.3. PPI Network Analysis and Screening of Hub Genes

**Figure 3:**
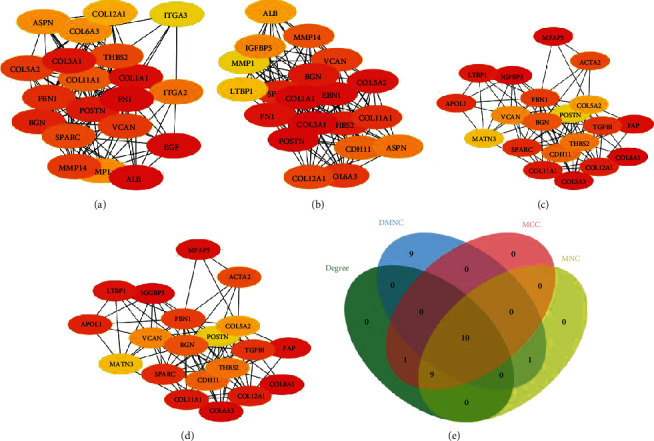
Identification of hub genes. (a–d) The hub genes were identified using four models (Degree, MCC, DMNC, and MNC) with the Cytoscape plugin cytoHubba. (e) A Venn diagram was used to identify the ten hub genes in PDAC.

To explore the potential interactions between the DEGs, we used the STRING database to study the relationship between the various DEGs. We identified ten hub genes based on the Degree (Figure 3(a)), MCC (Figure 3(b)), DMNC (Figure 3(c)), and MNC (Figure 3(d)) Cytoscape models. The ten hub genes were BGN, COL5A2, COL6A3, COL11A1, COL12A1, FBN1, POSTN, SPARC, THBS2, and VCAN (Figure 3(e)).

### 3.4. Validation of Expression and Alteration of Hub Genes in PDAC

**Figure 4:**
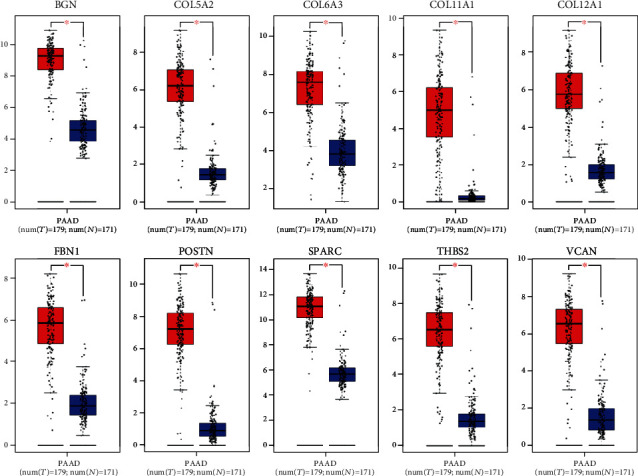
Expression analysis of ten hub genes in PDAC based on GEPIA. The mRNA expression levels in TCGA pancreatic tumors (*n* = 179) and matching normal tissue (*n* = 171) from the TCGA and GTEx databases. *P* < 0.05 was considered statistically significant.

**Figure 5:**
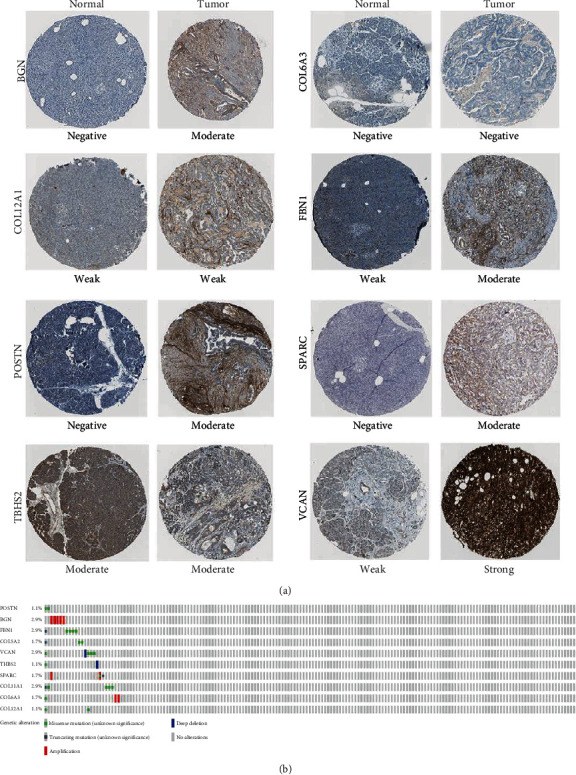
Validation of protein expression and genetic alterations of ten hub genes in pancreatic cancer. (a) Representative protein expression of eight genes in pancreatic cancer tumors and normal tissue. Data was obtained from the Human Protein Atlas database. (b) Genetic alterations of ten genes in pancreatic cancer. Data was obtained from the cBioPortal.

To verify the differential expression of the hub genes between PDAC and normal pancreatic tissue, we analyzed the ten hub genes using the GEPIA-based TCGA database. We found that the mRNA expression levels of the hub genes were significantly increased in PDAC compared to normal pancreatic tissue (Figure 4). Protein expression levels were similarly compared using the Human Protein Atlas database. Typical immunohistochemistry images (https://www.proteinatlas.org/) for the protein expression of eight of the ten genes (COL5A2 and COL11A1 were not included in the database) in tumor and normal pancreatic tissue are shown in Figure 5(a). Genetic alterations found in the ten genes in pancreatic cancer are shown in Figure 5(b). Missense mutations were the most common type of mutation in the upregulated genes.

### 3.5. Identification of the Clinical Prognostic Value of Hub Genes in PDAC

**Figure 6:**
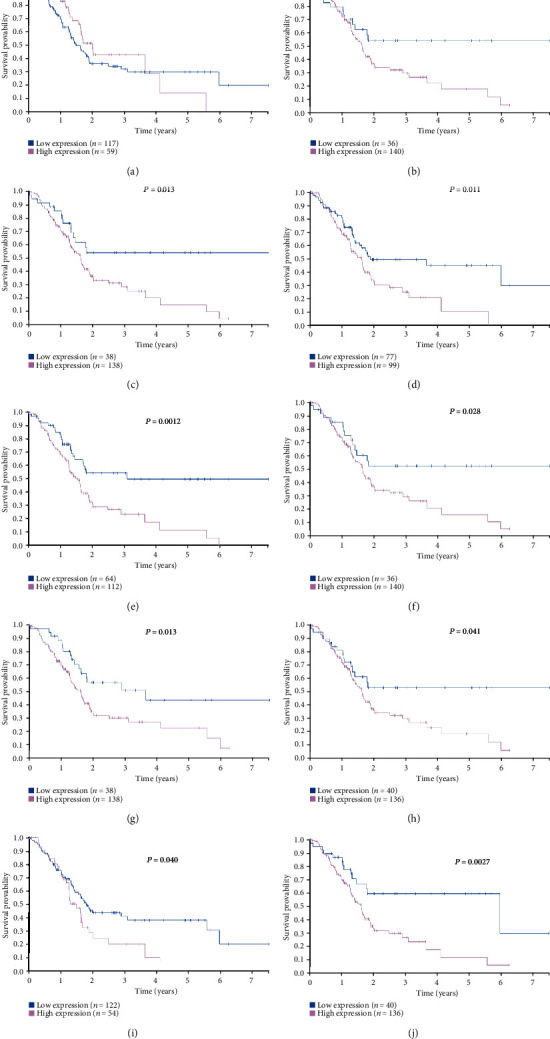
Survival analysis of the ten hub genes in PDAC based on the Human Protein Atlas. (a) BGN; (b) COL5A2; (c) COL6A; (d) COL11A1; (e) COL12A1; (f) FBN1; (g) POSTN; (h) SPARC; (i) THBS2; (j) VCAN. *P* < 0.05 was considered statistically significant.

To estimate the influence of hub gene expression on the prognosis of PDAC, we performed survival analysis for the hub genes using the Human Protein Atlas online tool for differential analysis. High expression of nine of the hub genes was positively correlated with poor prognosis (COL5A2 *P* = 0.044; COL6A3 *P* = 0.013; COL11A1 *P* = 0.011; COL12A1 *P* = 0.0012; FBN1 *P* = 0.028; POSTN *P* = 0.013; SPARC *P* = 0.041; THBS2 *P* = 0.040; VCAN *P* = 0.0027) (Figure 6).

### 3.6. Immune Infiltration Analysis

**Figure 7:**
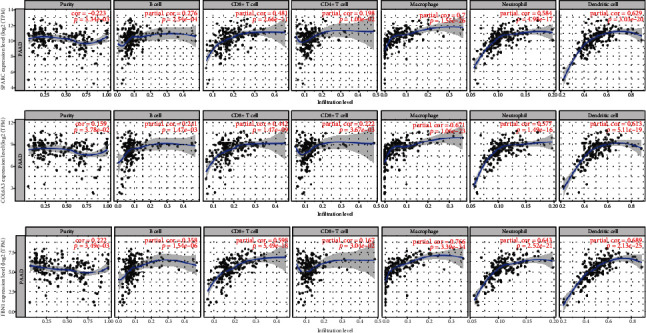
Correlation analysis of prognosis-related genes with tumor-infiltrating immune cell types using TIMER.

To explore whether there was a correlation between immune cell infiltration into the tumors and hub gene expression, we analyzed the relationships between the ten hub gene signatures and tumor purity and six important immune cell types (CD4+ T cells, CD8+ T cells, B cells, neutrophils, macrophages, and dendritic cells) using TIMER. We observed that most of these prognosis-related genes were positively correlated with the infiltrating levels of the different immune cell types but negatively related to tumor purity (Supplementary Figure 1, Supplementary Table 1). In particular, SPARC, COL6A3, and FBN1 showed remarkable positive correlations with the infiltrating levels of the six immune cell types (Figure 7).

**Figure 8:**
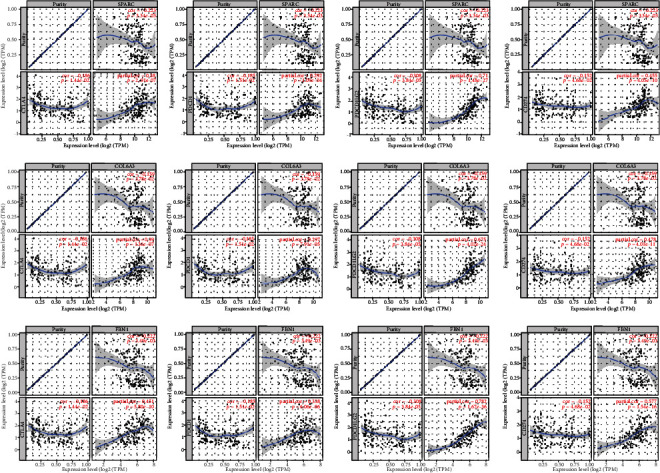
The association between the expression levels of SPARC (a), COL6A3 (b), and FBN1 (c) with immune checkpoints using TIMER.

Since immunotherapy is currently focused on immunological checkpoint inhibitors (e.g., CTLA4, PDCD1, PDCD1LG2, and CD274), we chose SPARC, COL6A3, and FBN1 as potential target genes and further analyzed the coexpression relationship of these three genes with these immune checkpoint-related genes by TIMER. We found that all three of these potential target genes had strong coexpression relationships with CTLA4, PDCD1, PDCD1LG2, and CD274 (Figure 8).

## 4. Discussion

Pancreatic cancer is one of the most malignant and aggressive tumors. Traditional treatment methods (e.g., surgery, chemotherapy, radiotherapy, and other locoregional therapies) provide low survival rates. Currently, several clinical studies have focused on immunotherapeutic strategies in pancreatic cancer [20]. Therefore, a better understanding of the immune infiltration into pancreatic tumors and the identification of novel PDAC immune-related biomarkers may prove useful for immunotherapy.

In the current study, we found 212 reliable DEGs in PDAC by the comprehensive analysis of three datasets. These differentially expressed genes were then subjected to GO and KEGG pathway enrichment analysis, which indicated that these DEGs were mainly enriched in pathways involved in the extracellular matrix (ECM) and extracellular structure organization and the PI3K-Akt signaling pathway. The ECM, a major component of the tumor microenvironment, is known to undergo significant changes during angiogenesis and tumor progression [21]. Activation of the PI3K-AKT signaling pathway can enhance pancreatic cancer cell proliferation and invasion [22, 23]. Our results can help elucidate the underlying mechanisms of TME in pancreatic cancer proliferation and invasion. Furthermore, we identified ten hub genes through PPI network analysis. According to the results of our prognostic analysis, high expression of COL5A2, COL6A3, COL11A1, COL12A1, FBN1, POSTN, THBS2, SPARC, or VCAN was associated with poor prognosis for patients with pancreatic cancer. These results suggest that these genes could serve as potential prognostic biomarkers for PDAC.

Immune cell infiltration in the tumor microenvironment has received much attention and has become a promising therapeutic target. To further investigate the relationship between these prognosis-related genes and different immune cell types, we predicted gene-immune cell interactions using TIMER. We found that the expression levels of SPARC, COL6A3, and FBN1 were significantly correlated with six infiltrating immune cell types (CD4+ T cells, CD8+ T cells, B cells, neutrophils, macrophages, and dendritic cells).

SPARC is a multifunctional calcium-binding glycoprotein that is usually secreted into the extracellular matrix and plays a key role in proliferation, migration, adhesion, and differentiation [24, 25]. Previous studies have shown that SPARC is localized in the tumor stroma and overexpressed in various cancers, such as breast [26], lung [27, 28], and melanoma [29]. Moreover, SPARC expression is dramatically increased in gastric tumors and associated with poor outcome [30]. Stromal SPARC expression is observed in almost 40% of pancreatic adenocarcinoma patients who have undergone curative resection, and this expression is an independent prognostic factor [31]. Consistent with our research, high SPARC expression in PDAC is associated with poor prognosis.

COL6A3 is an extracellular matrix protein that is typically found in most connective tissues, including muscle, skin, tendon, and vessels. Many recent studies have demonstrated the important role of COL6A3 in the diagnosis and prognosis of colorectal, lung, and prostate cancer [32, 33]. A high level of expression of COL6A3 has been observed in pancreatic tumors, where it was correlated with negative prognostic factors [34]. This finding is consistent with the results obtained in our study.

FBN1 encodes fibrillin, which is the primary component of microfibrils in the extracellular matrix. A previous study reported that FBN1 overexpression plays a key role in the development of germ cell tumors [35]. FBN1 is also a target gene for microRNA- (miR-) 133b. miR-133b inhibits the proliferation, migration, and invasion of gastric cancer cells by increasing FBN1 expression [36]. Moreover, hypermethylated FBN1 is found in tissue samples from colorectal cancer patients but not in healthy controls, suggesting that hypermethylated FBN1 may be a sensitive biomarker for this disease [37]. The role of FBN1 in the development of pancreatic cancer and its correlation with the prognosis of patients has not been reported previously. In summary, we speculate that SPARC, COL6A3, and FBN1 might be involved in the development of PDAC by regulating the functions of the TME.

Previous studies have suggested that the tumor microenvironment is one of the significant factors determining the prognosis of PDAC and may even play a role in the resistance to treatment [38, 39]. However, the tumor microenvironment is complex and determined by many factors. We comprehensively analyzed the correlation between immune checkpoints and identified three potential prognosis-related genes (SPARC, COL6A3, and FBN1). We were surprised to find that these genes had a significant coexpression relationship with CTLA4, PDCD1, PDCD1LG2, and CD274. These results suggest that the three prognosis-related genes may affect the development of pancreatic cancer by affecting immune checkpoints. Future studies should identify the specific roles of these genes in the regulation of PDAC development and progression.

## 5. Conclusion

Taken together, our results suggest that the nine high expression hub genes (COL5A2, COL6A3, COL11A1, COL12A1, FBN1, POSTN, THBS2, SPARC, and VCAN) may be associated with the development and prognosis of PDAC. All these genes may be potential prognostic biomarkers for PDAC. In addition, three hub genes (SPARC, COL6A3, and FBN1) may also play a vital role in the microenvironment of pancreatic cancer through the regulation of tumor-infiltrating immune cells, suggesting they could serve as potential therapeutic targets for the modulation of the antitumor immune response. Nevertheless, further investigation is needed to confirm the mechanisms underlying the potential roles of these biomarkers in the immune microenvironment.

## Data Availability

The data used to support the findings of this study are available from the corresponding author upon request.

## Supplementary Materials

Supplementary 1 Supplementary Figure 1: correlation analysis of the expression levels of BGN, COL5A2, COL11A1, COL12A1, POSTN, THBS2, and VCAN with tumor-infiltrating immune cell types using TIMER.

Supplementary 2 Supplementary Table 1: relationship between 10 hub gene expression and immune infiltration level in PDAC.
