# Supplementary figures and images for: Identification of Key Prognostic Biomarker and Its Correlation with Immune Infiltrates in Pancreatic Ductal Adenocarcinoma
Source: Dis Markers. 2020 Aug 31;2020:8825997. doi: 10.1155/2020/8825997 (PMC7479484; doi:10.1155/2020/8825997)

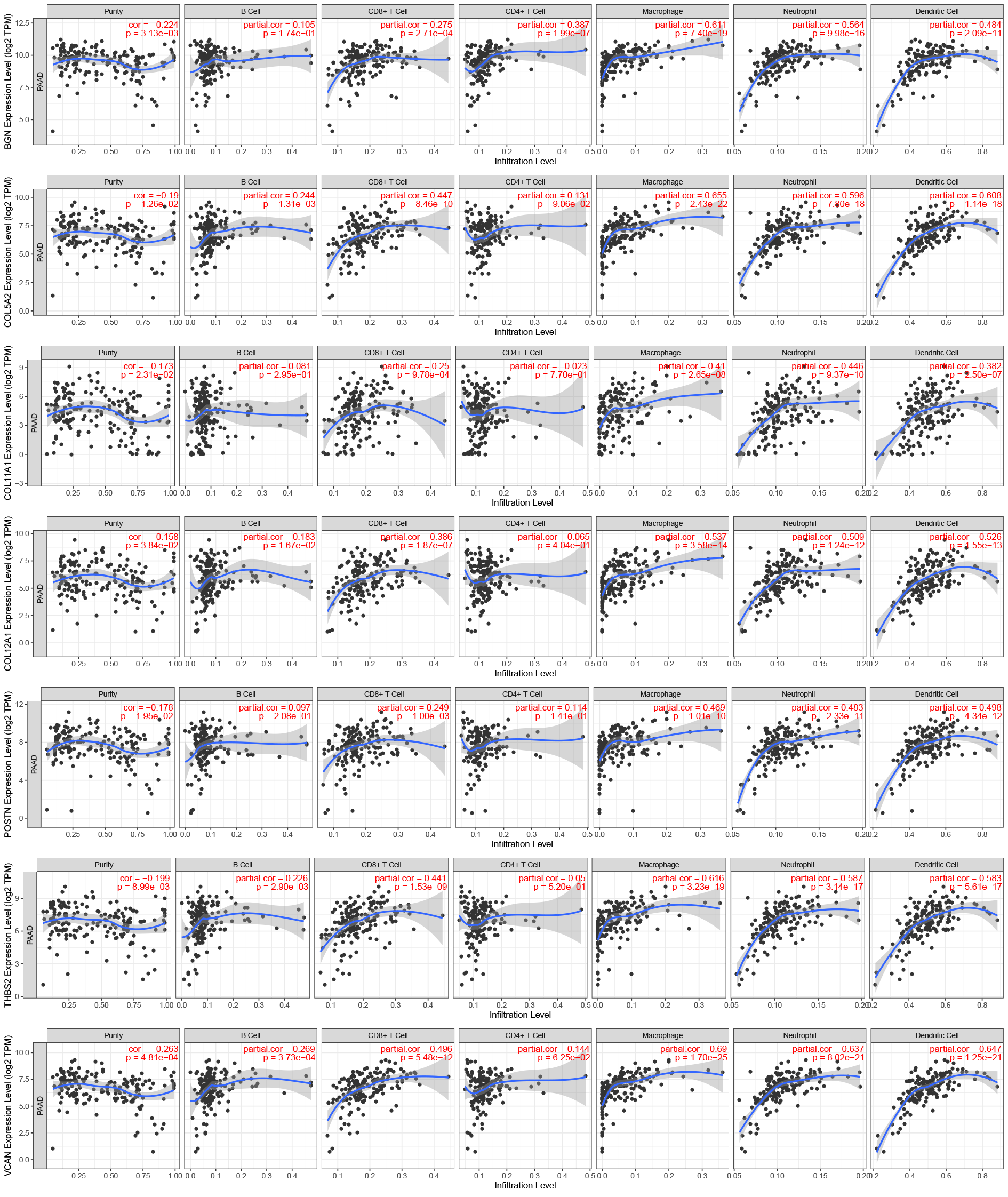

Supplement: Supplementary 1 — Supplementary Figure 1: correlation analysis of the expression levels of BGN, COL5A2, COL11A1, COL12A1, POSTN, THBS2, and VCAN with tumor-infiltrating immune cell types using TIMER. [file 8825997.f1.tif]
